# Coping with a chronic condition that requires lifelong medication: a qualitative study with people living with atrial fibrillation in São Paulo, Brazil

**DOI:** 10.1136/bmjopen-2024-088226

**Published:** 2025-06-09

**Authors:** Elisabete Paschoal, Rodrigo D Olmos, Tiffany E Gooden, Paulo A Lotufo, Isabela Bensenor, Kate Jolly, Gregory Y H Lip, G Neil Thomas, Sheila Greenfield, Deirdre Lane, Alessandra C Goulart, on behalf of the NIHR Global Health Research Group on Atrial Fibrillation Management, Ajini Arasalingam

**Affiliations:** 1 Center for Clinical and Epidemiological Research, Universidade de Sao Paulo, Sao Paulo, Brazil; 2 Universidade de São Paulo, Sao Paulo, Brazil; 3 Institute of Applied Health Research, University of Birmingham, Birmingham, UK; 4 ELSA Brasil Cohort Study, Sao Paulo, Brazil; 5 University of Birmingham, Birmingham, UK; 6 Liverpool Centre for Cardiovascular Science, University of Liverpool, Liverpool, Merseyside, UK; 7 Department of Epidemiology, School of Public Health, Universidade de Sao Paulo, Sao Paulo, Brazil

**Keywords:** Adult cardiology, Cardiovascular Disease, Aged

## Abstract

**Abstract:**

**Objective:**

To provide insight into how people cope with living with atrial fibrillation (AF) and taking oral anticoagulants (OACs), informing how services and healthcare delivery could be improved to offer the appropriate support patients require, thereby optimising their quality of life and well-being.

**Design:**

A qualitative study employing focus group discussions (FGDs).

**Setting:**

11 primary care units in a socioeconomically deprived area of the Butantan district in São Paulo, Brazil.

**Participants:**

Adults (≥18 years) with AF purposively recruited based on sex, age and socioeconomic status.

**Results:**

Saturation was met with three FGDs comprising seven, five and five participants, respectively. Theme one focused on self-management, where many participants discussed their methods for adhering to dietary restrictions and alternative medications, including plant-based options and specific foods, and how they modified their daily activities to reduce AF complications and symptoms. Theme two was rationality, where participants described three main ways that they cope with taking long-term medication (often warfarin): thinking that it controls their AF symptoms; it is an obligation; it prevents morbidity and premature death. Theme three was attitude and emotions, where participants described their initial reactions of shock and fear after diagnosis and ongoing emotions of sadness and frustration due to required self-management activities and regular blood tests. Theme four was medication regimen, where participants discussed difficulties with polypharmacy, changes to AF medication (particularly from non-vitamin K antagonist OACs (NOACs) to warfarin), side effects from taking warfarin and various methods of medication management.

**Conclusions:**

This study presents three key findings with implications for patient care and support. First, the shock and fear experienced during diagnosis due to a lack of knowledge about AF suggests that improvements in public knowledge about AF are needed. Second, people with additional chronic conditions may need improved care and support, given the concern participants had regarding when and how to take their medications safely. Third, improved access to NOACs may reduce the difficulties, frustrations and concerns participants had regarding warfarin use (eg, diet, dose adjustments, self-management and monthly international normalised ratio tests).

**STRENGTHS AND LIMITATIONS OF THIS STUDY**

- Despite the small sample size (n=17) inherent in qualitative studies, the sample was diverse, comprising individuals with atrial fibrillation (AF) who remained in care, representing patients from 11 out of 13 primary care units within the area and achieving data saturation.
- Although participants were from one area of Brazil, thus limiting transferability, many of our findings align with research from existing evidence (including in high-income countries), indicating that the care and support desired may represent a universal need.
- Only people with AF retained within the healthcare system were recruited; their experiences of coping with living with AF may systematically differ from people not retained in care.

## Introduction

 People living with a chronic condition often experience a cycle of hope and frustration regarding treatment, with expectations about treatment effectiveness and medical advice fluctuating throughout the course of their illness.^1^,^3^ For some conditions, people may experience what some sociologists term ‘the Sisyphus syndrome’ where patients try a treatment only to find minimal improvement in the condition or symptoms, resulting in the need to start over again with a new kind of treatment or dose of medication^1^; the impact of this can be physically and emotionally exhausting. Therefore, patients must adapt to, or cope with, their chronic condition and the potential changes in symptoms and medication over time. Coping with chronic long-term conditions, such as atrial fibrillation (AF), can be defined as processes an individual learns and does regarding how to tolerate the effects of illness.^1^ Qualitative evidence on how patients cope with AF, a chronic condition that requires daily medication, lifestyle modifications and often monthly blood tests, is lacking, particularly in low- and middle-income countries (LMICs).

AF is the most common arrhythmia globally and increases the risk of stroke 5-fold,^4^ although oral anticoagulants (OACs) can significantly reduce the risk of stroke.^5 6^ Vitamin K antagonists such as warfarin are commonly prescribed in LMICs, but these require periodic testing to measure the international normalised ratio (INR).^57^,^9^ Additionally, the warfarin dose requires adjustments, and the patient may also need to make changes to diet and lifestyle behaviours (eg, reducing alcohol intake, avoiding contact sports, etc).^10^ While non-VKA OACs (NOACs) have similar efficacy compared with warfarin^11^,^13^ and do not require INR testing, they are less readily available in LMICs.^14 15^ Thus, people with AF in LMICs have little option but to manage their condition with warfarin and monthly blood tests with regular dose adjustments.

Evidence suggests that living with AF and experiences with poor INR control adversely affect quality-adjusted life years.^16^ A 2017 systematic review found six qualitative studies that reported on patients’ attitudes towards and perceptions of OACs; although all were conducted in high-income countries (HICs).^17^ From the six themes identified,^17^ the data suggested that AF patients felt they were often misinformed about the effects of OACs from other patients, caregivers and physicians, had diverse views on the benefits and risks of OACs and experienced difficulties in implementing the doctors’ instructions in the daily management of medication. Patients also emphasised their dissatisfaction with the difficulties and costs related to INR monitoring, their desire for more shared decision-making between them and their doctors, and the impact that AF had on their daily living, including limitations and restrictions to diet and exercise.^17^ Since this review, another qualitative study was conducted, which investigated patient perspectives on OAC treatment among 10 patients with AF from Australian general practices. Authors reported the following key facilitators: patient passive attitudes regarding decision-making, adequate explanation on health advice from doctors for better prognosis and patients having a clear understanding of OACs. Two key barriers were sometimes the lack of explanation from doctors and the inconvenience of taking warfarin.^18^

These findings contribute to our knowledge of patients’ views on OACs; however, it must also be understood how patients cope with the diagnosis, responsibilities and self-care required to effectively manage AF, particularly in LMICs where barriers to adequate healthcare and the ability for self-care often differ to those reported in HICs.^17^ Through focus group discussions (FGDs) strategy, we aimed to provide insight into how AF patients cope living with this condition in Brazil, to inform services and healthcare delivery from an LMIC setting about the appropriate support patients require to optimise their quality of life and well-being.

## Methods

### Study design

This qualitative study used FGDs to elicit patients’ experiences of coping with AF.^19^ The data were collected as part of a larger multicountry mixed-methods study aimed at identifying and assessing the AF care pathway,^20^ as part of the National Institute for Health and Care Research (NIHR) Global Health Research Group on AF management.^21^ Recruitment for the FGDs commenced in February 2020 but was interrupted due to the COVID-19 pandemic; FGDs resumed and were completed in November 2021.

### Study setting and participants

This study was conducted in a socioeconomically deprived area of the Butantan district in São Paulo, Brazil. Brazil is an upper middle-income country^22^ with a well-established universal healthcare system where healthcare visits and basic medications and treatments are available free of charge to all citizens.^23^ São Paulo, located in the southeast, is the most populated city in Brazil. The Butantan district of São Paulo has a population of more than 540 000 residents^24^ and an estimated 2.4% prevalence of AF in adults aged 65 years or older.^25^

There are 15 primary care units (PCUs) in Butantan. Participants were recruited from the 11 PCUs connected with the University of São Paulo. Patients were eligible if they were aged 18 years or older, had a confirmed AF diagnosis and had received their AF diagnosis or AF management care (including INR tests and medications refills) in one of the 11 included PCUs. Patients were recruited purposively based on sex, age and socioeconomic status, with the aim of reaching data saturation^26^ with three FGDs. There were no exclusion criteria.

### Data collection

FGDs were conducted in a private room within the Hospital Universitário of the University of São Paulo and audiorecorded to permit verbatim transcription. The location was selected for its proximity and ease of access for patients in the Butantan area. Participants were seated at a round table and joined by members of the research team (EP, ACG). EP (female) facilitated the FGDs; EP is a trained anthropologist with extensive experience in collecting qualitative data. ACG (female) assisted with facilitating the interviews; ACG is a medical doctor trained in qualitative methods. A female nurse also attended the FGDs to take notes on participants’ body language, question comprehension and interactions with one another. No member of the research team performing the FGDs had a prior relationship with the participants.

The topic guide (online supplemental material 1) was developed collaboratively by researchers in the UK and Brazil. Questions in the topic guide covered patients’ experience of being diagnosed with AF, taking and adhering to AF medication, side effects and symptoms from AF or AF medication and the impact of living with AF on day-to-day activities. The FGDs were carried out in the local language of Brazilian-Portuguese and were audiorecorded and transcribed verbatim in Brazilian-Portuguese by an external specialist (with a confidentiality agreement in place). Transcripts were then translated into English by an experienced bilingual speaker within the NIHR Global Health Research Group on AF Management.^21^

### Data analysis

Data were independently analysed by two medical doctors (ACG and RDO), the same anthropologist (EP) who facilitated the discussions and a UK-based global health researcher (TEG) trained in qualitative methods. We conducted a content analysis, a well-known method for FGDs.^27^ Initially, the transcripts were open coded by reviewing all text line by line and applying descriptive codes to words, sentences and paragraphs that were related to patient actions and the emotions patients felt due to AF. Axial coding was carried out in the next step of the process to reduce the descriptive codes by repackaging and combining the codes to identify subthemes and themes.^28^ Regular meetings with the research team, which included qualitative experts (SG, DL), occurred throughout the analysis process to discuss and agree on the final themes and subthemes.

### Ethical considerations and reporting standards

The Ethics Committee from the Hospital Universitário of the University of São Paulo approved the study protocol (approval number: 94732318.6.0000.0076). All participants provided written informed consent prior to recording and commencing each FGD. We report our qualitative study following the Standards for Reporting Qualitative Research checklist for mixed-methods studies (online supplemental file 1: Topic Guide).^29^

### Patient and public involvement

There was no patient or public involvement in the development of this study.

## Results

20 patients were invited to take part; however, three declined. The remaining 17 patients provided written consent to participate in one of three FGDs (seven, five and five participants, respectively).^30^ The median age of participants was 58 years (IQR: 53.5–66.5 years), nine were men, most had completed secondary education (n=11), were married (n=13) and were either retired (n=6) or unable to work (n=5) (table 1). Our data met saturation, with no new themes arising from the third and final FGD.

**Table 1:** Participant characteristics

Characteristic	Focus group 1n=7	Focus group 2n=5	Focus group 3n=5
Age group			
40–60 years	3	3	3
61+years	4	2	2
Sex			
Female	2	2	4
Male	5	3	1
Educational level			
Did not complete primary school	1	0	1
Completed primary school	2	1	0
Completed secondary school	4	4	1
Undergraduate degree	0	0	2
Unknown	0	0	1
Marital status			
Single	1	0	0
Married	4	5	4
Divorced	1	0	1
Widowed	1	0	0
Employment status			
Employed	1	2	2
Housewife	0	0	1
Retired	4	0	2
Unable to work	2	3	0

**Figure 1:**
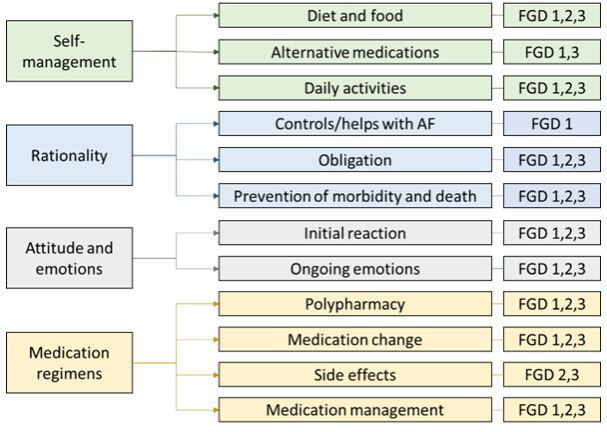
The coding tree for the main themes captured from the three FGDs. FGD, focus group discussion.

The coding tree (figure 1) depicts four main themes that were captured from the FGDs: self-management, rationality, attitude and emotions and medication regimens. Each theme and its corresponding subthemes are described below in more detail with supported quotes; additional quotes from the FGDs can be found in the supplementary material (online supplemental table S1). Most subthemes were mentioned in all FGDs; however, where this was not the case, the FGDs in which the theme arose are noted.

### Theme 1: self-management

The first theme of *self-management* comprised three subthemes of *diet and food, alternative medications* and *daily activities*. Through *diet and food,* participants discussed what they ate and what foods they avoided to stay healthy, prevent complications and adhere to the doctors’ instructions. The avoidance of green vegetables and limiting foods rich in vitamin K was raised in all three FGDs, particularly from participants on warfarin. Some participants discussed their technique for rationing their intake of green vegetables to ensure consumption of a safe yet healthy amount. However, these participants were from a low-middle income area of São Paulo with traditions that embrace lots of vegetable consumption. One participant found the management of *diet and food* due to AF difficult because they wanted to be vegetarian but were advised against it, given the restrictions they were given on green vegetable consumption.

I don't eat anything green because of Warfarin … not like kale, stuff like that. They say it changes (my INR control), right? Mine changes if I eat. (FGD2, P4)

They don't want to allow me to become a vegetarian… and to become a vegetarian I would have to eat a lot of dark vegetables, but as I take Warfarin, I can't. (FGD1, P1)

The use of *alternative medicines* for *self-management* was raised in the first and third FGD, mainly regarding the use of plants and certain foods for medicinal purposes. The use of Jatoba was commonly mentioned, which can be consumed as fruit or flour from the bark of Jatoba trees. Additionally, participants mentioned the use of garlic, herbal tea and leaves from Ginkgo Biloba trees. Participants in the third FGD discussed medications they use or do not use for *self-management,* including which pain medicines to use based on possible interactions with warfarin.

I started taking ‘Jatobá’ peel because I got prostate cancer and since I took it the cancer has disappeared. And garlic, garlic is very good for blood pressure. (FGD1,P1)

some precautions you have to take because of that, like I take a dipyrone, I can't take Paracetamol, there’s aspirin, I can't take it, as well. Taking warfarin has the contraindication of taking care to take any other medication you take. (FGD 3, P3)

Due to warfarin use, participants from all three FGDs raised many examples of how they developed and changed *daily activities* for the purpose of *self-management*. To protect themselves against the risks of injuries and bleeding, participants said they stopped participating in certain hobbies, changed where they went and how they exercised and became more vigilant about items they lifted or being around any sharp items. Participants felt these new *daily activities* were necessary to avoid complications or the worsening of their condition; however, it often came at the cost of not doing the things they once enjoyed.

It affected me because I can't pick up weight anymore, I know that depending on the injury, you must be wise, the most impactful was the weight. (FGD2, P2)

What I was sad about was that I liked riding a motorcycle and my kids forbade me to ride. They were afraid that I would be involved in an accident and die, because I didn't have time to be helped because of the haemorrhage. (FGD3, P2)

### Theme 2: rationality

Three subthemes, *controls/helps with AF, obligation and prevention of morbidity and death, resulted from the second theme of rationality* (online supplemental table S1). Despite the restrictions caused by using warfarin, a few participants from the first FGD recognised the importance of adhering to the long-term use of medication in terms of *controlling and helping with AF,* although they recognised that it would not cure AF.

Warfarin makes it easier for your heart to pump. (FGD1, P7)

Whereas participants from all three FGDs viewed taking warfarin as an *obligation,* many insinuated that they have no alternatives to the medication and that it is just a part of life. They declared acceptance and compliance with the guidance from health professionals.

It’s warfarin, it’s another thing, ‘you gotta do what you gotta do’ (FGD 2, P5)

Most participants in all FGDs mentioned that the consistent use of warfarin is necessary for the *prevention of morbidity and death*, particularly from stroke. Participants mentioned that adhering to warfarin helps them to survive, even if taking warfarin means restrictions on *diet and food* and *daily activities*. Some admitted that they did not have a full understanding of the magnitude of benefits from taking warfarin, but they knew it reduced their risk of death and therefore they adhered.

As I understand it, I had an ischaemic stroke, started ischaemic then turned into haemorrhagic. Then I take warfarin to prevent clots from forming in the bloodstream, thereby preventing me from having a new stroke. (FGD1, P1)

I don't know much about medicine, I just take care not to die. (FGD3, P2)

### Theme 3: attitude and emotions

The third theme of *attitude and emotions* had two distinct subthemes identified from all FGDs: *initial reaction* and *ongoing emotions* (online supplemental table S1). For many, the diagnosis was unexpected, and some had never heard of AF, which initially caused anxiety, shock and fear in some participants. However, most participants explained that they were reassured following discussions with the doctor, and particularly after finding the right warfarin dose to take. Although participants did not welcome the news of their AF diagnosis, many accepted it quite easily after becoming more knowledgeable about the condition and the medication required to manage it.

I was worried, but after I did the tests, the doctor explained to me and I started taking the medication … after that I enjoyed life, I hanged out… Before I thought it was not possible, but I can do my things … so I continued to have a good life, well, normal life. (FGD3, P5)

As participants adapted to living with AF, which required daily medication and frequent INR tests, their *ongoing emotions* were variable. Many participants mentioned the sadness they felt due to the *obligation* of taking warfarin every day. Other participants spoke about being fearful and worried that their *self-management* of *diet and food* and *daily activities* were inadequate to avoid complications. One participant also expressed the annoyance of having to take regular INR tests, while another participant expressed their gratitude for warfarin despite any difficulties, limitations or challenges that they experienced.

I've never been on medication. I never liked it, not even for a headache. So, having to take this medicine every day, I am sad. (FGD1, P7)

[taking warfarin] you're afraid of getting hurt and a lot of blood coming out, will I have a haemorrhage? (FGD3, P4)

### Theme 4: medication regimen

The following four subthemes resulted from the *medication regimen* theme: *polypharmacy, medication change, side effects* and *medication management* (online supplemental table S1). Participants from all three FGDs spoke of comorbidities and how they take multiple medications in addition to warfarin. Due to this *polypharmacy,* some participants expressed difficulties and uncertainties about when they should take all their medications. While some participants expressed their acceptance of, and willingness to, live with their *medication regimen,* other participants did not understand why they must continue taking so many medications if their blood tests and examinations were good.

I've been doing these treatments, taking various medications, Propafenone, which is for the heartbeat, I take the medication for diabetes, metformin, I take warfarin, I take blood pressure medicine, the blood fat medicine… but I started to get intrigued because my tests are excellent, everything I do, the only thing that show on the tests is the arrhythmia. I asked him why almost all the tests are good and I still have to take a lot of medicine, so he said they are good because of the medicine. (FGD3, P2)

Participants from all FGDs discussed their journey of *medication change* to get to where they are now. Most participants were on warfarin at the time of the FGDs; however, a few participants had previous experience with NOACs for the management of AF. Participants preferred taking NOACs in terms of not having to take INR tests, but often they could not afford NOACs as they are not offered free of charge in Brazil and therefore, they were forced to switch to taking warfarin. Several participants spoke about their previous experience with aspirin and how it was not effective or made them feel unwell, which finally led to them switching to use warfarin.

But there are better medicines … Pradaxa [NOAC] they gave me, I didn't need to do [INR test], you take it, it’s very expensive … I'm working and everything, but it’s already difficult to pay … it’s very difficult, medicine, it shouldn't have that price. (FGD3, P1)

Once on warfarin, participants from the second and third FGD discussed the many *side effects* they experienced and the many *side effects* they are now attentive to, such as any signs of bleeding or clinical changes. Participants expressed their difficulties in adjusting and adhering to warfarin, but most accepted that it was part of their day-to-day life now. Many participants expressed difficulty in understanding how to take warfarin. Others said they were unable to monitor the INR because the tests are unavailable in the nearest health facility, and many faced problems with dietary restrictions due to warfarin.

Veins started to appear here in the left leg, bruises, this is visible, it really bothers me, if I put my hand on my leg it hurts, it becomes sensitive in the leg, in addition to being visible, this anticoagulant problem, which is a good and bad thing, if I don't take it anymore it can form a clot, give a heart attack, or a stroke, but I need the warfarin, I need the anticoagulant, if it doesn't thin the blood, it won't be able to help keep my heart balance its beat, so you see, those are unpleasant symptoms I'll have. (FGD3, P4)

*Medication management* can often be a challenge, especially for elderly patients; however, participants’ fear of forgetting or taking the wrong dosage led to them developing several strategies to remind themselves: some used an alarm clock to remind them of the medication; some separated the dosages in a box that identifies the time of day that each tablet should be taken; and some set reminders in strategic places. Despite these tactics, a few participants admitted to forgetting their dose or not taking the correct dose but expressed that it is something they try hard to avoid.

Veins started to appear here in the left leg, bruises, this is visible, it really bothers me, if I put my hand on my leg it hurts, it becomes sensitive in the leg, in addition to being visible, this anticoagulant problem, which is a good and bad thing, if I don't take it anymore it can form a clot, give a heart attack, or a stroke, but I need the warfarin, I need the anticoagulant, if it doesn't thin the blood, it won't be able to help keep my heart balance its beat, so you see, those are unpleasant symptoms I'll have. (FGD3, P4)

If you forget once in a while … that’s fine. So, for example, it has already happened to me: I went to take a shower, took my shower, laid in bed and the reminder came, then I was in doubt, did I take the medicine? As I have a stepdaughter who is a doctor, I called her saying that I had forgotten to take all my medications, she said that everything was fine and that tomorrow morning you will take them but avoid not forgetting. (FGD3, P2)

## Discussion

Our findings from three FGDs indicate that people with AF, as with other chronic long-term conditions, often go through a series of emotions and reactions following diagnosis in an attempt to cope with living with the long-term condition. Three key findings have implications for patient care and support. First, participants expressed feelings of shock and fear at the time of diagnosis due to a lack of knowledge about AF. Second, people with AF often have one or more comorbidities,^31^ and taking OACs alongside other medications was often raised as a concern for participants due to uncertainties about when and how to take their medications safely. Third, participants receiving warfarin expressed difficulties, frustrations, concerns, sadness and uncertainty regarding warfarin use, particularly relating to restrictions on diet, dose adjustments, self-management and monthly INR tests.

Previous qualitative studies have reported patients’ perceptions regarding the use of VKAs; however, most data are from HICs and do not capture indepth information about other aspects of coping with living with the chronic condition.^32^,^37^ Our findings that people lack information on AF before and after diagnosis are consistent with existing evidence,^34^,^37^ causing patients to feel fearful and distressed. Many of our participants were unaware of the causes, risks and management of AF; many did not understand why it was necessary to continue on warfarin if their INR was within the therapeutic range. The lack of knowledge contributed to the difficulties that some participants faced in finding a way to cope with the condition.

Better public awareness that AF is common and easily diagnosed and managed could aid in reducing fear or psychological distress following AF symptoms and diagnosis. Following diagnosis, it is crucial for patients to have access to the information they need to help them understand the aetiology of the condition, medication options and their associated risks and benefits, the management process and what they should or should not do to maintain good health and prognosis. Such information can reduce fear and psychological distress, encourage self-care and empower them to take part in the decision-making process for their treatment plan. Evidence-based information may reduce misunderstandings related to diet and other lifestyle behaviours mentioned within the FGDs, which have been said to originate from advice given by ill-informed community members in other settings.^34^,^36^ The effectiveness of standardised evidence-based information along with other opportunities to help patients with AF cope (eg, peer support) should be explored.^33^

Most participants in the present study had multiple comorbidities, which is common among people with AF.^31^ Living with multiple long-term conditions often has a negative impact on quality of life, clinical outcomes and, due to polypharmacy, the risk of drug-drug interactions is heightened.^38^ Managing multiple prescriptions can be complicated (eg, some medications must be taken with food, others at certain times of the day and others should be avoided with certain prescription or over-the-counter drugs), which can cause significant stress. Indeed, our findings revealed that people with AF have concerns regarding the uncertainties about when and how to take the many medications they have alongside warfarin. AF is managed in secondary care within specialised anticoagulation clinics in Brazil and many other LMICs^14 39^; this care is often fragmented and separate from clinics providing care for other long-term conditions, creating challenges for people with multiple long-term conditions and healthcare professionals that manage these patients. To ensure patient safety, improve clinical outcomes and reduce polypharmacy, information continuity between and within healthcare facilities is critical.^40^ This can be done through integrating services (electronically and/or physically) and/or patient-held documents. However, our previous research indicated that patient-held documents are not well used in Brazil for AF (manuscript in preparation), and most facilities are often not electronically mature enough for integration,^39^ indicating the need for strengthening healthcare infrastructure and systems to ensure people with AF have the information and advice they need for managing multiple long-term conditions simultaneously.

Warfarin is free of charge in the Brazilian public healthcare system, but patients must pay out of pocket for NOACs. Some participants mentioned how they preferred taking NOACs but were forced to take warfarin due to the costs of NOACs; a finding consistent with other qualitative studies.^34^,^36^ This indirectly created challenges for many people with AF in our study. Regular INR tests, as required when on warfarin,^41^ were difficult to maintain for people with AF due to the lack of local infrastructure to perform these tests, the costs and time involved with travelling to clinics or hospitals and the overall burden of attending the healthcare facility monthly. Similar findings have been reported from other qualitative studies conducted in HICs with people with AF and their caregivers.^34^,^37^ From previous pathway mapping of AF care in Brazil,^39^ Sri Lanka^42^ and China (under review), access to and regular need for INR tests were reported as barriers to optimal AF care. Furthermore, a previous study reported that primary care clinicians often feel less confident about prescribing and monitoring warfarin due to uncertainties about bleeding risk.^43^ Healthcare professionals in Brazil have indicated their preference for prescribing NOACs over warfarin for the majority of people with AF,^44^ and NOACs could reduce the need for INR tests and therefore reduce visits to healthcare facilities, which for many people in LMICs require a long distance to travel. NOACs could, in turn, improve clinical care for people with AF and their ability to cope with living with AF, as well as reduce costs to patients and healthcare systems. However, NOACs are not suitable for all patients with AF (those with mechanical heart valves or moderate-to-severe mitral stenosis), and OAC choice should be made on a case-by-case basis, considering risk factors, patient preferences and drug availability.^10^

Using NOACs could also reduce the frustrations caused by diet restrictions on warfarin, a finding highlighted from nearly all qualitative studies on AF,^33^,^36^ including the present study. There is no evidence that one should refrain from eating foods rich in vitamin K when taking VKA such as warfarin.^45^ Anecdotally, however, physicians often recommend that people with AF reduce their intake of foods high in vitamin K, which includes green vegetables such as spinach, cabbage and broccoli. The underlying rationale for this lies in the action mechanism of warfarin that interferes with the action of vitamin K and therefore prolongs the time it takes to form a clot, the intended effect of anticoagulant therapy.^44^ Increasing vitamin K intake while you are on warfarin may therefore work against the action of this medication.^44^ Although this is not a strict recommendation, a systematic review by Violi *et al*^45^ found that people with AF believe that avoidance of vitamin K-rich foods is a requirement when on warfarin and their knowledge about diet is incorrect. Particularly in Brazil, we have reported previously that patients’ beliefs regarding the interference of a vitamin K-rich diet on coagulation are a key concern.^46^ Efforts should be made to change how this recommendation is communicated in practice, particularly in LMICs where warfarin is used more frequently and plant-based diets are more prevalent.^14^

### Strengths and limitations

Our study included people with AF from 11 of the 15 primary and secondary care facilities in the Butantan area. Although our sample was small with 17 participants, our sample included a diverse group of people with AF retained in care within the area, and data saturation was met. Although participants were from one area of Brazil, thus limiting transferability, many of our findings align with research from existing evidence (including in HICs), indicating that the care and support desired may represent a universal need.

## Conclusions

This qualitative study aimed to provide insight on how patients with AF cope with living with the condition in Brazil to inform services and healthcare delivery from an LMIC setting about the appropriate support patients require to optimise patients’ quality of life and well-being. Our findings highlight that people with AF often feel shocked and worried about their initial diagnosis due to lack of knowledge about AF, feel burdened with the inconvenience of monthly INR tests when on warfarin, feel worried about how to take warfarin in conjunction with other medications and feel forced to take warfarin due to the inaccessibility of NOACs and uncertainty regarding daily lifestyle behaviours, such as diet. Improvements in public knowledge about AF, accessibility of NOACs and improved care and support for people with multiple chronic conditions may be warranted to aid people with AF cope better with living with this chronic condition in a way that reduces stress and optimises well-being and quality of life.

## Supplementary material

10.1136/bmjopen-2024-088226 online supplemental file 1

10.1136/bmjopen-2024-088226 online supplemental file 2

## Data Availability

Data are available in a public, open access repository. All data relevant to the study are included in the article or uploaded as supplementary information.
