# Supplementary material for: Coping with a chronic condition that requires lifelong medication: a qualitative study with people living with atrial fibrillation in São Paulo, Brazil
Source: BMJ Open. 2025 Jun 9;15(6):e088226. doi: 10.1136/bmjopen-2024-088226 (PMC12161368; doi:10.1136/bmjopen-2024-088226)
Supplement: online supplemental file 1 [file bmjopen-15-6-s001.docx]

**TOPIC GUIDE FOR PATIENTS FOCAL GROUP ON AF AND ANTICOAGULATION**

>> WARNING: turn-on the recorders (2)

**I. WARM UP/ INTRODUCTION:**

"*Hello…. My name is 'name' and I'm here to coordinate our discussions today ... Thanks for coming. We are recording our discussion just to analyze and compare it with other groups, ok?”*

• **Important:** **ask participants to introduce themselves briefly using the name they would like to be called during the discussions (they should already be written on cards / badges)**: "*Before we start, I would like everyone to be able to introduce themselves to the group using the name you would like to be called during our meeting today ".*

• **Appoint someone to start the process and then go over each member of the group**.

• **Thank the group for their introduction, define the ground rules (see the ground rules in the focus group setup document) and start with the first question.**

**II. INTRODUCTION TO THE AF THEME:**

1. “*Do you know what Atrial Fibrillation is? Who could explain it to me? There are no doctors here… so, please explain in your own words…. "*

2. *'We have invited you because you have Atrial Fibrillation, or a heart problem and we would like to hear your views on some aspects of this problem'* ...

**III.GENERAL UNDERSTANDING AND PERCEPTIONS ABOUT AF**

**ESSENTIAL QUESTIONS**

1. Can anyone tell me a little about how you found out you had this heart problem?

>> **Try to encourage all group members to tell their story ... did this happen to anyone else? Does anyone else have a different experience?**
2*. What did the doctor / nurse tell you about this problem?***>> Allow patients to discuss freely first and then, if necessary, use specific questions to gain an understanding of the different problems and perceptions they mentioned:** *a) mechanism: the heart does not work properly, does the heart beat irregularly / very quickly?
b) treatment: that is, curable / treatable?*

3. When you were told you had AF, how did you feel?

*>>* ***QUESTIONS TO INCLUDE IF YOU HAVE TIME / PARTICULAR INTEREST IN YOUR COUNTRY***

4. Has anyone received information about what the doctor / nurse's procedure would be like?

*>>* **Ask for details if the initial answer is just "yes"**.

5. Did anyone look for information on their own?

>> **For example, internet, books, brochures, talking to other people, other healthcare professionals, websites.**
6. What do you think causes AF?

>> **for example, other medical conditions, lifestyle behaviors (smoking, alcohol, drugs, obesity).**

7. Can anyone tell me what the symptoms of AF are? What did you feel?

>> **for example, tiredness and less ability to exercise; shortness of breath; feeling faint or dizzy, chest pain >> some people do not have any symptoms (this is normal), so it is important to explain whether they had symptoms.**
8. Did you tell your family / friends / co-workers about this health problem?

*>>* **Depending on the answers, check why / why not?**

**III. AF MANAGEMENT**

“*We explored how you discovered you had AF, along with your understanding and views on the disease. Before we move on, is there anything that anyone would like to add? I would now like to ask you a few questions about how you deal AF.”*

>> **ESSENTIAL QUESTIONS**

9. *Can you tell me about the different treatments you have done for AF?*

>> ***If they talk about anticoagulation and this is not discussed, ask****:*

a) What do you take (ORAL ANTICOAGULANT / WARFARINA / ETC)?
b) Why do you take it? What are the benefits?

>> **They should talk about reducing the risk of stroke here >> also ask about other used / known treatments**

>> **Explore why they take antiarrhythmic drugs, why they had catheter ablation or cardioversion, etc.**

>> **If they are not getting any anticoagulants, why? (personal choice etc.)**

10. *Doctors prescribe anticoagulants (medicine to reduce the risk of blood clots) to control atrial fibrillation - can someone tell me what this medicine is and how it works?* >> **After the first answer, try to get an understanding of other people, if no answer offers a correct explanation, that is, dilute the blood, ask other people's opinion**.

>> **If there are incorrect answers, it is also important to provide an explanation to ensure that group members know what it is about correctly. Check if the participants say they were taking it and stopped because it was: the doctors’ decision, own decision, side effects, bleeding etc.**

11. *In addition to other treatments you have had, do you do anything else to try to control AF?*

>> **Possible questions may be losing weight, change diet, stop smoking / reduce alcohol intake, exercise, control blood pressure, complementary / alternative medicine, vitamins / supplements**

12. *Do you think that atrial fibrillation can put people at risk of developing other medical problems?*

>> **Stroke, heart failure.**
>> **The facilitator needs to say at this point: "For the next questions, if you are not on anticoagulation treatment, think about how you would feel if you were."**

13. *How did you feel when / if your doctor prescribed / or were to prescribe anticoagulant medications for you?*
14. *Can you tell me what medications you are taking and if you are happy to take them?* >> **Ask about the side effects they have had if they do not come naturally, such as "Has anyone had side effects as a result of the anticoagulant medication they are taking? Or did they have side effects that led them to stop the anticoagulant? "**
>> **Note that if the patients mention specific medications in response to question 9, the facilitator may ask: 'Did you tell me earlier that you take medication X and medication Y and take any other medications? ... if they say yes, ask why?**15. *Do you normally remember to take your anticoagulant medication? Have you ever forgotten?*
16. *Do you think it matters / does it matter if you take the medication every day or not?*
17. *What do you think can happen if you take more medications or less medications than prescribed?*
18. *How does AF affect your daily life? How do these symptoms affect your quality of life?* 
19. *How does anticoagulation treatment affect your daily life?*

>> **NOTE: It will probably be important to ask the previous two questions separately to try to clarify whether it is the disease or the medication or both that have the greatest impact on the patient's life**

>> **EXPLORE: quality of life, side effects, presentation of regular symptoms, impediment of the patient to do everyday things, need to take care of himself, need to modify the diet / alcohol consumption, need to go to the doctor more often for check-up, causes side effects when feeling anxious / makes the patient feel safe about the risk of stroke.**

>> **QUESTIONS TO INCLUDE IF YOU HAVE TIME OR PARTICULAR INTEREST IN YOUR COUNTRY:**

20. *How long have you been informed that you should continue taking your anticoagulant? (weeks, months or years)*

**IV. TREATMENT JOURNEY**

*"We talked a little bit at the beginning about how you found out you had AF .... I would like to explore a little more now"*
>> **ESSENTIAL QUESTIONS**

21. *Please explain to me which health professionals you consulted when you were diagnosed with your heart problem ... please, can you tell me about your individual stories from diagnosis to the present day?*

>> **If no one responds to this, you could give a possible example >> for example, you saw your general practitioner who then referred you to the cardiologist ... he sent you to do the X exam ... then he returned / took a certain medication.**

>> **QUESTIONS TO INCLUDE IF YOU HAVE TIME OR PARTICULAR INTEREST IN YOUR COUNTRY**22. *How often do you have consultations for the treatment of atrial fibrillation and what kind of health professionals and visits to clinics or hospitals do you need to make?*

>> **If no one answers that, you can give a possible example, for example: I go to clinic x every month or every 6 months to do a blood test and then they write to my family doctor, and I get my prescription from my family doctor… Or just go to my herbalist (?) once a month and get a mixture of herbs to boil and drink as tea….**

**V. AF AND TREATMENT INFORMATION**
>> **ESSENTIAL QUESTIONS**
23. *When you go to the doctor / clinic for a check-up, do you receive notes or written documents to return to the clinic the next time or hand them over to another doctor when you are referred to see someone else?*
**IF YES for Q23:**

a) *What is the nature of these documents / pieces of paper? What do these documents contain?*

>> **For example, prescriptions, comments about the visit to the clinic / discharge from treatment / prescription of anticoagulants ...**
b) *What do you think of the written information / documents you have?*

*>>* **for example, it is useful for you / helped you understand your health conditions / AF / the importance of taking medications as instructed by your doctors.**
c) *How do you use them?*

>> **for example, consult them at home, show them to other people, take them to the next appointments.**
d) *How do you try to keep them safe / guarded?*

>> **QUESTIONS TO INCLUDE IF YOU HAVE TIME OR PARTICULAR INTEREST IN YOUR COUNTRY**
24. *If you have any questions about AF and your medication, where or with whom do you seek answers?*25. *Is there anything else you would like to have to help you understand and manage your heart problem better?*

>> **If nothing is mentioned, indicate how things could improve, for example: written information / website address for consultation / advice / professional healthcare support .**

>> **NOTE: At the end of the focus group, it is important to thank the participants for their contributions and give them the opportunity to say anything additional that they consider relevant and that was not part of the discussion already held:**

*“Thank you all for your contribution today. Before concluding, is there anything relevant that you think we haven't covered or anything else that you would like to talk about / add?"*
