# Supplementary material for: Coping with a chronic condition that requires lifelong medication: a qualitative study with people living with atrial fibrillation in São Paulo, Brazil
Source: BMJ Open. 2025 Jun 9;15(6):e088226. doi: 10.1136/bmjopen-2024-088226 (PMC12161368; doi:10.1136/bmjopen-2024-088226)
Supplement: online supplemental file 2 [file bmjopen-15-6-s002.docx]

Supplementary Table S1. Supporting quotes for each theme and sub-theme

| **Themes and sub-themes** | **Supporting quotes (FGD number; Participant number)** |
| --- | --- |
| **Theme 1: Self-management** |  |
| **Diet and food** | “They don't want to allow me to become a vegetarian... and to become a vegetarian I would have to eat a lot of dark vegetables, but as I take Warfarin, I can't.” (FGD1, P1) |
|  | “I don't eat anything green because of Warfarin ... not like kale, stuff like that. They say it changes (my INR control), right? Mine changes if I eat.” (FGD2,P4) |
|  | “green vegetables if you eat today, let's assume you will eat 100 grams today, all. One day you will have to eat 100 grams, as if it were medication, so as not to shock your body with information … but if you eat an amount, you always have to eat the same amount, all your life, in a week you eat 100 grams, you have to eat the whole week 100 grams.” (FGD3,P3) |
| **Alternative medicines** | “I started taking “Jatobá” peel because I got prostate cancer and since I took it the cancer has disappeared. And garlic, garlic is very good for blood pressure.”(FGD1,P1) |
|  | “some precautions you have to take because of that, like I take a dipyrone, I can't take Paracetamol, there's aspirin, I can't take it, as well. Taking warfarin has the contraindication of taking care to take any other medication you take.” (FGD 3,P3) |
| **Daily activities** | “You have to be careful for not to cut yourself because you end up bleeding too much. And I didn't have that before. Now, a little glass that falls on your leg makes it to start to bleed.” (FGD1, P5) |
|  | “It affected me because I can't pick up weight anymore, I know that depending on the injury, you must be wise, the most impactful was the weight.” (FGD2,P2) |
|  | “What I was sad about was that I liked riding a motorcycle and my kids forbade me to ride. They were afraid that I would be involved in an accident and die, because I didn't have time to be helped because of the haemorrhage.” (FGD3,P2) |
| **Theme 2: Rationality** |  |
| **Controls/helps with AF** | “Warfarin makes it easier for your heart to pump.” (FGD1,P7) |
|  | “Warfarin only helps.” (FGD 1,P2) |
| **Obligation** | “I am a person who respects the professional a lot, when you are a professional and you pass something on to me, you did it because you know what you are doing, so I received it very naturally, you have to take [warfarin], you have to take it” (FGD3,P2) |
|  | “It’s warfarin, it’s another thing, ‘you gotta do what you gotta do’” (FGD 2, P5) |
| **Prevention of morbidity and death** | “As I understand it, I had an ischemic stroke, started ischemic then turned into haemorrhagic. Then I take Warfarin to prevent clots from forming in the bloodstream, thereby preventing me from having a new stroke.” (FGD1,P1) |
|  | “I don't know much about medicine, I just take care not to die.” (FGD3, P2) |
|  | “[Warfarin], as I understand it, it is an anticoagulant, so it prevents you from creating a clot. I can't have clots because of the valve, I can't because I already had a stroke. As far as I understand this.” (FGD2,P4) |
|  | “[warfarin] helping me to survive, but I must be careful, of course, not everything we consume we like, but it's necessary, so in the face of need I like it, it's nice to me, it helps me to survive.” (FGD3,P4) |
| **Theme 3: Attitude and emotions** |  |
| **Initial reaction** | “I was worried, but after I did the tests, the doctor explained to me and I started taking the medication ... after that I enjoyed life, I hanged out… Before I thought it was not possible, but I can do my things … so I continued to have a good life, well, normal life.” (FGD3,P5) |
|  | “I already knew warfarin, I already knew, but I never imagined that I would have to take it. I know the first thing I said to the doctor was how long I would have to take it and he said it would be for the rest of my life… so I didn't like it, but I accepted, because it's a question of my survival.” (FGD2,P4) |
| **Ongoing emotions** | “I've never been on medication. I never liked it, not even for a headache. So, having to take this medicine every day, I am sad.” (FGD1,P7) |
|  | “My biggest fear is warfarin, because it has vitamin K, green stuff has a lot of vitamin K, so they affect the way warfarin works.” (FGD2,P4) |
|  | “I would feel happy if I had full health and didn't have to take anything, but on the other hand I feel happy to have this alternative that helps keep me going.” (FGD3,P2) |
|  | “[taking warfarin] you're afraid of getting hurt and a lot of blood coming out, will I have a haemorrhage?” (FGD3,P4) |
| **Theme 4: Medication regimens** |  |
| **Polypharmacy** | “I just take it, of course I must take it, but I take 5 pills in the morning, 1 at lunch and 1 at dinner. Among my pills in the morning is warfarin, 1 pill, it's what I take a day, I feel good about it.” (FGD2,P1) |
|  | “When I came from the hospital, I took it at five in the morning, which was the time they had at the hospital, but it was very bad for me to take it because then I had my Omeprazole, I also had to take levothyroxine, that was all very complicated, so I talked to the doctor for me to start taking it at night, it's the last thing I do.” (FGD 2,P4) |
|  | “I've been doing these treatments, taking various medications, Propafenone, which is for the heartbeat, I take the medication for diabetes, Metformin, I take warfarin, I take blood pressure medicine, the blood fat medicine… but I started to get intrigued because my tests are excellent, everything I do, the only thing that show on the tests is the arrhythmia. I asked him why almost all the tests are good and I still have to take a lot of medicine, so he said they are good because of the medicine.” (FGD3,P2) |
| **Medication change** | “I take warfarin. At first, I took Rivaroxaban, but as it was very expensive, I asked to change it and today I only take warfarin. This happened when I was 51 years old. I took Rivaroxaban for about 3, 4 months. And I have been taking warfarin for 3 years.” (FGD1,P5) |
|  | “This warfarin I've been taking for 20 years. Before, I don't know what I took, it was an anticoagulant…I don’t remember the name, but some doctors over the years [stopped my anticoagulant]… it made me feel bad after the time not taking it, because the blood thickened and I ate the greens that thickened the blood and I took aspirin, but I think the aspirin was a little pill and it was not enough, but I started again from 2019 until now.” (FGD3,P4) |
|  | “But there are better medicines … Pradaxa (NOAC) they gave me, I didn't need to do [INR test], you take it, it's very expensive … I'm working and everything, but it's already difficult to pay … it’s very difficult, medicine, it shouldn't have that price.” (FGD3,P1) |
| **Side effects** | “I took the dose he prescribed, there was his prescription, a dose, after 30 days I was hospitalized because my INR was 18, my urine was already brown, I had tremendous pain, I had to stay in the hospital, from then on I had to control and then I saw how dangerous it is.” (FGD2,P4) |
|  | “Veins started to appear here in the left leg, bruises, this is visible, it really bothers me, if I put my hand on my leg it hurts, it becomes sensitive in the leg, in addition to being visible, this anticoagulant problem, which is a good and bad thing, if I don't take it anymore it can form a clot, give a heart attack, or a stroke, but I need the warfarin, I need the anticoagulant, if it doesn't thin the blood, it won't be able to help keep my heart balance its beat, so you see, those are unpleasant symptoms I'll have.” (FGD3,P4) |
| **Medication management** | “First of all, don't forget the medicine, you can't forget it. You must take the medicine every day, right; if you forget, you will suffer.” (FGD1,P2) |
|  | “yesterday I was supposed to have taken one and a half, but I took one, since I take it straight up, today I took one and a half, because it's Tuesday and Friday one and a half, on the other days one. So, these little things, when they increase, it gets in the way a little, I write, I try to write, now I'll put it right in front of the fridge because I forgot yesterday, I had to take it today” (FGD2,P5) |
|  | “I'm super organized with these things; I have a kit where all the medicines are there. In each little window I write what it is, so I know what it is, what time I should take it. I also have an App that is “Medsafe”, it starts rattling when I take the medicine and it looks like a medicine box, it says the time to take the medicine, then I go there and take it.” (FGD3,P2) |
|  | “If you forget once in a while … that's fine. So, for example, it has already happened to me: I went to take a shower, took my shower, laid in bed and the reminder came, then I was in doubt, did I take the medicine? As I have a stepdaughter who is a doctor, I called her saying that I had forgotten to take all my medications, she said that everything was fine and that tomorrow morning you will take them, but avoid not forgetting.” (FGD3,P2) |
