# Supplementary material for: Coping with a chronic condition that requires lifelong medication: a qualitative study with people living with atrial fibrillation in São Paulo, Brazil
Source: BMJ Open. 2025 Jun 9;15(6):e088226. doi: 10.1136/bmjopen-2024-088226 (PMC12161368; doi:10.1136/bmjopen-2024-088226)
Supplement: Supplementary file 3 [file bmjopen-2024-088226.reviewer_comments.pdf]

PEER REVIEW HISTORY

BMJ Open publishes all reviews undertaken for accepted manuscripts. Reviewers are asked to complete a checklist review form and are provided with free text boxes to elaborate on their assessment. These free text comments are reproduced below.

This paper was submitted to another journal from BMJ but declined for publication following peer review. The authors addressed the reviewers' comments and submitted the revised paper to BMJ Open. The paper was subsequently accepted for publication at BMJ Open.

ARTICLE DETAILS

Title (Provisional)

Coping with a chronic condition that requires lifelong medication: A qualitative study with people living with atrial fibrillation in São Paulo, Brazil

Authors

Paschoal, Elisabete; Olmos, Rodrigo D; Gooden, Tiffany E; Lotufo, Paulo A; Bensenor, Isabela; Jolly, Kate; Lip, Gregory YH; Neil Thomas, G.; Greenfield, Sheila; Lane, Deirdre; Goulart, Alessandra C.; Atrial Fibrillation Management, NIHR Global Health Research Grou

---

VERSION 1 - REVIEW

**Reviewer**	**1**
**Name**	**Reviewer 1**
**Affiliation**	
**Date**	**18-Apr-2024**
**COI**	**No competing interest**

Comments not published pending reviewer's consent

**Reviewer**	**2**
**Name**	**Reviewer 2**
**Affiliation**	
**Date**	**18-Apr-2024**
**COI**	**No competing interest**

Comments not published pending reviewer's consent

VERSION 2 - REVIEW

**Reviewer**	**3**
**Name**	**Gebreyohannes, Eyob Alemayehu**
**Affiliation**	**The University of Western Australia, Pharmacy**
**Date**	**01-Aug-2024**
**COI**	**None.**

This study evaluated how people cope with living with atrial fibrillation and taking oral anticoagulants through three focus group discussions involving 17 participants. The manuscript is also well-written and offers valuable insights into the challenges patients with atrial fibrillation face, especially from an LMIC perspective.

I would like to congratulate the authors for their excellent work. I only have some comments and minor suggestions.

TITLE

I suggest the following minor change to the title "Coping with a chronic condition requiring lifelong medication: Focus group discussions with people living with atrial fibrillation in São Paulo, Brazil"

ABSTRACT

The statement "Empirical evidence on how people cope with a chronic condition requiring daily medications is lacking." is a bit misleading. As the authors stated in the 'What is already known on this topic' section, there are studies from high-income countries. Hence, I suggest rephrasing the sentence to indicate the lack of/limited evidence from LMICs.

WHAT THIS STUDY ADDS:

• The expression 'People', 'Many people', etc is inaccurate as this study included a handful of people. Instead, I suggest phrasing it as, along the lines, 'Our participants with AF…', 'Most of our participants…' etc.

• "…the corresponding restrictions…": delete "the corresponding".

HOW THIS STUDY MIGHT AFFECT RESEARCH, PRACTICE OR POLICY

• First line of the third bullet: comma after 'that'

INTRODUCTION

Page 7 Line 4: I suggest changing the phrase "medical advice rising and falling…" to improve clarity.

Page 7 Line 12-14: Do you mean empirical evidence is lacking from LMICs? Please clarify. Also, it would be important to describe what the literature from HICs says to provide some context (e.g., 10.1186/s12875-021-01453-5 and 10.3390/pharmacy11050153).

Page 7 Line 17: Is warfarin the most commonly used OAC? Is this consistent across all countries, including both HICs and LMICs? I am uncertain if this statement is accurate. In many HICs, NOACs have become the most commonly prescribed OACs for patients with AF. This distinction needs to be clarified in the introduction.

RESULTS

Page 13 Line 2: Delete the comma after 'restrictions'.

Page 14 Line 3-4: I suggest rephrasing the sentence to improve clarity. E.g., "Most participants in all FGDs mentioned that the consistent use of warfarin is necessary for the prevention of morbidity and death, particularly from stroke."

REPORTING CHECKLIST

The authors stated that they have used the COREQ checklist. It should be uploaded as a supplementary file.

**Reviewer**	**4**
**Name**	**Spark, M. Joy**
**Affiliation**	**University of New England, Pharmacy**
**Date**	**24-Feb-2025**
**COI**	**None**

Thank you for the opportunity to review this interesting manuscript, for what appears to be a qualitative descriptive study. Below are some ideas for how the manuscript could be improved.

The abstract provides a good summary of the text, and the conclusion should be reframed to address the stated aim of providing insights into how people cope.

The opening paragraph of the introduction has a single reference from 1991. Are there other, more current, authors who have something to say on this topic?

The objective, at the end of the introduction, could be simplified to what is achievable from a qualitative study and separated from possible uses of the data.

The methods were generally comprehensive but not able to be replicated because the supplementary material 1 referred to was missing.

The narrative would be improved if the participants voices could be heard more clearly by incorporating the quotes into the results section text rather than including them in a separate table.

There appears to be some misreferencing on page 20 of 32. Reference 24 to 26 mentioned in line 18 are methodology references rather than included studies and Line 20 references 35 and 38 appear to be the same, and a reference relating to China in the 35,38,39 group missing. It would be good to check all reference citations are accurate.

The claim, in Line 22 on the same page, about Healthcare professionals is supported by a reference about healthcare professionals in Brazil, so this restriction should be mentioned, or references from other countries added to support the claim.

Some of the strengths and limitations mentioned, such as small sample sizes, are integral to qualitative research. One of the limitations not mentioned is that the people were from one area of Brazil so the results may not reflect other areas of Brazil or other countries.

The conclusion should directly address the aim/objectives of the study, with the other ideas, particularly the first sentence of the current conclusion, incorporated into the discussion.

How people cope with AF is a valuable topic, thank you for investigating it.

VERSION 2 - AUTHOR RESPONSE

Reviewer 3 Comments:

Comment #1:
This study evaluated how people cope with living with atrial fibrillation and taking oral anticoagulants through three focus group discussions involving 17 participants. The manuscript is also well-written and offers valuable insights into the challenges patients with atrial fibrillation face, especially from an LMIC perspective. I would like to congratulate the authors for their excellent work. I only have some comments and minor suggestions.

Reply to Comment #1: Thank you for the positive assessment and useful feedback. We hope you find our response below and that the corresponding amendments have elevated the manuscript.

Comment #2*: TITLE: I suggest the following minor change to the title "Coping with a chronic condition requiring lifelong medication: Focus group discussions with people living with atrial fibrillation in São Paulo, Brazil"*

Reply to Comment #2: Thank you for this suggestion. We have amended the title following the Editor´s request as well. It now reads as follows: "Coping with a chronic condition that requires lifelong medication: A qualitative study with people living with atrial fibrillation in São Paulo, Brazil."

Comment #3: *ABSTRACT: The statement "Empirical evidence on how people cope with a chronic condition requiring daily medications is lacking." is a bit misleading. As the authors stated in the 'What is already known on this topic' section, there are studies from high-income countries. Hence, I suggest rephrasing the sentence to indicate the lack of/limited evidence from LMICs.*

Reply to Comment #3: Thank you for this. Also, following the Editor's request, we have restructured the abstract, which resulted in this text being deleted.

Comment #4: *WHAT THIS STUDY ADDS:*
• The expression 'People', 'Many people', etc is inaccurate as this study included a handful of people. Instead, I suggest phrasing it as, along the lines, 'Our participants with AF…', 'Most of our participants…' etc.
• "…the corresponding restrictions…": delete "the corresponding".

Reply to Comment #4: The Editor requested that this section be deleted and that the study's 'Strengths and limitations' section be added to follow the abstract; therefore, this text has been deleted.

Comment #5: *HOW THIS STUDY MIGHT AFFECT RESEARCH, PRACTICE OR POLICY*
• First line of the third bullet: comma after 'that'

Reply to Comment #5: As mentioned above, this section has been deleted to be in line with BMJ Open´s instructions

Comment #6: *INTRODUCTION: Page 7, Line 4: I suggest changing the phrase "medical advice rising and falling…" to improve clarity.*

Reply to Comment #6: Thank you for this suggestion. We have amended this text to clarify that we are referring to the back-and-forth of experiencing both positive and negative feelings. See modifications below:

"People living with a chronic condition often experience a cycle of hope and frustration regarding treatment, with expectations about treatment effectiveness and medical advice fluctuating throughout the course of their illness." (Page 6, lines 2-4)

Comment #7: *Page 7 Line 12-14: Do you mean empirical evidence is lacking from LMICs? Please clarify. Also, it would be important to describe what the literature from HICs says to provide some context (e.g., 10.1186/s12875-021-01453-5 and 10.3390/pharmacy11050153).*

Reply to Comment #7: In this sentence, we were referring specifically to the lack of qualitative studies, particularly in LMICs. Thank you for sharing the study from Australia. We have now added this reference and have amended the introduction as below.

*"Qualitative evidence on how patients cope with AF, a chronic condition that requires daily medication, lifestyle modifications and often monthly blood tests, is lacking, particularly in low- and middle-income countries (LMICs). (*Page 6, lines 12-14)

"*Since this review, another qualitative study was conducted, which investigated patient perspectives on OAC treatment among 10 patients with AF from Australian general practices. Authors reported the following key facilitators: patient passive attitudes regarding decision-making, adequate explanation on health advice from doctors for better prognosis, and patients having a clear*

understanding of OACs. Two key barriers were sometimes the lack of explanation from doctors and the inconvenience of taking warfarin.[19]" (Page 6, lines 11-18)

Reference: 19. Gebreyohannes EA, Salter SM, Chalmers L, Radford J, Lee K, D'Lima D. Patients' Perspectives on Commencing Oral Anticoagulants in Atrial Fibrillation: An Exploratory Qualitative Descriptive Study. Pharmacy. 2023 Sep 23;11(5):153.

Comment #8: *Page 7 Line 17: Is warfarin the most commonly used OAC? Is this consistent across all countries, including both HICs and LMICs? I am uncertain if this statement is accurate. In many HICs, NOACs have become the most commonly prescribed OACs for patients with AF. This distinction needs to be clarified in the introduction.*

Reply to Comment #8: Thank you for bringing this to our attention. We have amended this text and the references to clarify that vitamin K antagonists, such as warfarin, are most prescribed for AF in many low- and middle-income countries (LMICs) (see below). We also added an example from Brazil, where the public health system provides warfarin at no additional cost to patients, but NOACs are not free to patients. Two references from LMIC were added in this paragraph:

"AF is the most common arrhythmia globally and increases the risk of stroke 5-fold[2], although oral anticoagulants (OACs) can significantly reduce the risk of stroke. [3,4] Vitamin K antagonists such as warfarin are commonly prescribed in low and middle-income countries (LMICs), but these require periodic testing to measure the international normalized ratio (INR). Additionally, warfarin dose requires adjustments, and the patient may also need to make changes to diet and lifestyle behaviours (e.g., reducing alcohol intake, avoiding contact sports, etc.).[9] Whilst non-vitamin K OACs (NOACs) have similar efficacy compared to warfarin[10,11 ,12] and do not require INR testing, they are less readily available in LMICs. [15,16] In Brazil, for example, the public health system provides warfarin at no additional cost to patients with AF, but not NOACs.[13] Thus, people with AF in LMICs have little option but to manage their condition with warfarin and complete monthly blood tests with regular dose adjustments." (Page 6, lines 15-25)

References:

15. Santos IS, Goulart AC, Olmos RD, Thomas GN, Lip GY, Lotufo PA, Bensenor IM. Atrial fibrillation in low-and middle-income countries: a narrative review. European Heart Journal Supplements. 2020 Dec;22(Supplement_O):O61-77.

16. Bayer V, Kotalczyk A, Kea B, Teutsch C, Larsen P, Button D, Huisman MV, Lip GY, Olshansky B. Global Oral Anticoagulation Use Varies by Region in Patients With Recent Diagnosis of Atrial Fibrillation: The GLORIA-AF Phase III Registry. Journal of the American Heart Association. 2022 ;11(6):e023907.

Comment #9: *RESULTS: Page 13 Line 2: Delete the comma after 'restrictions'.*

Reply to Comment #9: We have removed the comma.

Comment #10: *Page 14 Line 3-4: I suggest rephrasing the sentence to improve clarity. E.g., "Most participants in all FGDs mentioned that the consistent use of warfarin is necessary for the prevention of morbidity and death, particularly from stroke."*

Reply to Comment #10: We have amended the sentence per your suggestion (as below).

"Most participants in all FGDs mentioned that the consistent use of warfarin is necessary for the prevention of morbidity and death, particularly from stroke." (Page 13, lines 1-2)

Comment #11: *REPORTING CHECKLIST The authors stated that they have used the COREQ checklist. It should be uploaded as a supplementary file.*

Reply to Comment #11: Apologies for not uploading this; however, the editor has asked for the SRQR checklist, and therefore, this is the reporting checklist we have now uploaded. We have changed this in our manuscript as well. See below:

"We report our qualitative study following the Standards for Reporting Qualitative Research (SRQR) checklist for mixed-methods studies (Supplementary file 1: Topic Guide).[30] "

Reference: 30. O'Brien BC, Harris IB, Beckman TJ, Reed DA, Cook DA. Standards for reporting qualitative research: a synthesis of recommendations. Acad Med. 2014;89(9):1245-1251.

Reviewer 4 Comments:

Comment #1:*Thank you for the opportunity to review this interesting manuscript, for what appears to be a qualitative descriptive study. Below are some ideas for how the manuscript could be improved.*

Reply to Comment #1: Many thanks for taking the time to review and provide useful suggestions to improve our manuscript. We hope you find our responses and manuscript amendments have adequately met your expectations.

Comment #2: *The abstract provides a good summary of the text, and the conclusion should be reframed to address the stated aim of providing insights into how people cope.*

Reply to Comment #2: Thank you for this suggestion. Please note the abstract has been restricted following the editor's comment, and we now have the objective of the study specifically highlighted at the start of the abstract (see below). We hope this now makes the aim of the study clearer in the abstract.

"Objectives: To provide insight into how people cope with living with atrial fibrillation (AF) and taking oral anticoagulants (OACs), informing how services and healthcare delivery could be improved to offer the appropriate support patients require, thereby optimizing their quality of life and well-being.
Design: A qualitative study employing focus group discussions (FGDs).
Setting: 11 primary care units in a socioeconomically deprived area of the Butantan district in São Paulo, Brazil.
Participants: Adults (≥18 years) with AF purposively recruited based on sex, age, and socioeconomic status.

Results: *Saturation was met with three FGDs comprising seven, five, and five participants, respectively. Theme one focused on self-management, where many participants discussed their methods for adhering to dietary restrictions and alternative medications, including plant-based options and specific foods, as well as how they modified their daily activities to reduce AF complications and symptoms. Theme two was rationality, where participants described three main ways that they cope with taking long-term medication (often warfarin): thinking that it controls their AF symptoms, it is an obligation, and it prevents morbidity and premature death. Theme three was attitude and emotions, where participants described their initial reactions of shock and fear after diagnosis and ongoing emotions of sadness and frustration due to required self-management activities and regular blood tests. Theme four was medication regimen, where participants discussed difficulties with polypharmacy, changes to AF medication (particularly from non-vitamin K antagonist OACs [NOACs] to warfarin), side effects from taking warfarin, and various methods of medication management.*
Conclusions: *This study presents three key findings with implications for patient care and support. First, the shock and fear experienced at the time of diagnosis due to lack of knowledge about AF suggests improvements in public knowledge about AF are needed. Second, people with additional chronic conditions may need improved care and support, given the concern participants had regarding when and how to take their medications safely. Third, improved access to NOACs may reduce the difficulties, frustrations and concerns participants had regarding warfarin use (e.g. diet, dose adjustments, self-management and monthly INR tests).“*

Comment #3: *The opening paragraph of the introduction has a single reference from 1991. Are there other, more current, authors who have something to say on this topic?*

Reply to Comment #3: Although this reference is dated, it is still a highly relevant and referenced paper in today’s research, given its original and impactful recommendations on using various terms (including ‘coping’) within sociological research regarding chronic illnesses. For instance, it was referenced 45 times within the last year. We have, therefore, kept this reference but also added two more recent references that either extend or apply this article’s original concept.

References:
2. Mahmoud K, Baker TA, Esiaka D, Balogun S. The need to appear healthy: Concealment of chronic illness, privacy, and self-sufficiency among chronically ill older Nigerians. Innovation in Aging. 2024;8(4):igad141.

3. Estecha Querol S, Clarke P, Sattler EL, Halford JC, Gabbay M. Non-visible disease, the hidden disruptive experiences of chronic illness in adversity. International journal of qualitative studies on health and well-being. 2020;15(1):1857579.

Comment #4: *The objective, at the end of the introduction, could be simplified to what is achievable from a qualitative study and separated from possible uses of the data.*

Reply to Comment #4: We reformulated the grammar of this part to clarify our aim better. We’re happy to amend if further details are provided. See below:

“Through focus group discussions (FGDs) strategy, we aimed to provide insight into how AF patients cope living with this condition in Brazil, to inform services and healthcare delivery from an LMIC setting about the appropriate support patients require to optimise their quality of life and well-being.” (Page 7, lines 22-24 and Page 8, lines 1-2)

Comment #5: *The methods were generally comprehensive but not able to be replicated because the supplementary material 1 referred to was missing.*

Reply to Comment #5: Apologies for this omission; we have now uploaded the supplementary materials, which include the topic guide (Supplementary file 1).

Comment #6: *The narrative would be improved if the participants voices could be heard more clearly by incorporating the quotes into the results section text rather than including them in a separate table.*

Reply to Comment #6: Thank you for this suggestion. We have now incorporated some quotes into the main document. See below (Pages: 12-19):

Theme 1: Self-management

The first theme of self-management comprised three sub-themes of diet and food, alternative medications, and daily activities (Table 2). Through diet and food, participants discussed what they ate and what foods they avoided to stay healthy, prevent complications and adhere to the doctors' instructions. The avoidance of green vegetables and limiting foods rich in vitamin K was raised in all three FGDs, particularly from participants on warfarin. Some participants discussed their technique for rationing their intake of green vegetables to ensure consumption of a safe, yet healthy, amount. However, these participants were from a low-middle income area of São Paulo with traditions that embrace lots of vegetable consumption. One participant found the management of diet and food due to AF difficult because they wanted to be vegetarian but were advised against it, given the restrictions they were given on green vegetable consumption.

"I don't eat anything green because of Warfarin ... not like kale, stuff like that. They say it changes (my INR control), right? Mine changes if I eat." (FGD2, P4)
"They don't want to allow me to become a vegetarian... and to become a vegetarian I would have to eat a lot of dark vegetables, but as I take Warfarin, I can't." (FGD1, P1)

The use of alternative medicines for self-management was raised in the first and third FGD, mainly regarding the use of plants and certain foods for medicinal purposes. The use of Jatoba was commonly mentioned, which can be consumed as fruit or flour from the bark of Jatoba trees. Additionally, participants mentioned the use of garlic, herbal tea and leaves from Ginkgo Biloba trees. Participants in the third FGD discussed medications they use or do not use for self-management, including which pain medicines to use based on possible interactions with warfarin.

"I started taking "Jatobá" peel because I got prostate cancer and since I took it the cancer has disappeared. And garlic, garlic is very good for blood pressure." (FGD1,P1)
"some precautions you have to take because of that, like I take a dipyrone, I can't take Paracetamol, there's aspirin, I can't take it, as well. Taking warfarin has the contraindication of taking care to take any other medication you take." (FGD 3, P3)

Due to warfarin use, participants from all three FGDs raised many examples of how they developed and changed daily activities for the purpose of self-management. To protect themselves against the risks of injuries and bleeding, participants said they stopped participating in certain hobbies, changed where they went and how they exercised and became more vigilant about items they lift or being around any sharp items. Participants felt these new daily activities were necessary to avoid

complications or the worsening of their condition; however, it often came at the cost of not doing the things they once enjoyed.

"It affected me because I can't pick up weight anymore, I know that depending on the injury, you must be wise, the most impactful was the weight." (FGD2, P2)
"What I was sad about was that I liked riding a motorcycle and my kids forbade me to ride. They were afraid that I would be involved in an accident and die, because I didn't have time to be helped because of the haemorrhage." (FGD3, P2)

Theme 2: Rationality

Three sub-themes, controls/helps with AF, obligation and prevention of morbidity and death, resulted from the second theme of rationality (Table 2). Despite the restrictions caused by using warfarin, a few participants from the first FGD recognised the importance of adhering to the long-term use of medication in terms of controlling and helping with AF; although they recognized that it would not cure AF.

"Warfarin makes it easier for your heart to pump." (FGD1, P7)

Whereas participants from all three FGDs viewed taking warfarin as an obligation; many insinuated that they have no alternatives to the medication and that it is just a part of life. They declared acceptance and compliance with the guidance from health professionals.

"It's warfarin, it's another thing, 'you gotta do what you gotta do'" (FGD 2, P5)

Most participants in all FGDs mentioned that the consistent use of warfarin is necessary for the prevention of morbidity and death, particularly from stroke. Participants mentioned that adhering to warfarin helps them to survive, even if taking warfarin means restrictions on diet and food and daily activities. Some admitted that they did not have a full understanding of the magnitude of benefits from taking warfarin, but they knew it reduced their risk of death and therefore they adhered.

"As I understand it, I had an ischemic stroke, started ischemic then turned into haemorrhagic. Then I take Warfarin to prevent clots from forming in the bloodstream, thereby preventing me from having a new stroke." (FGD1, P1)
"I don't know much about medicine, I just take care not to die." (FGD3, P2)

Theme 3: Attitude and emotions

The third theme of attitude and emotions had two distinct sub-themes identified from all FGDs: initial reaction and ongoing emotions (Table 2). For many, the diagnosis was unexpected, and some had never heard of AF which initially caused anxiety, shock and fear in some participants. However, most participants explained that they were reassured following discussions with the doctor, and particularly after finding the right warfarin dose to take. Although participants did not welcome the news of their AF diagnosis, many accepted it quite easily after becoming more knowledgeable about the condition and medication required to manage it.

"I was worried, but after I did the tests, the doctor explained to me and I started taking the medication ... after that I enjoyed life, I hanged out… Before I thought it was not possible, but I can do my things … so I continued to have a good life, well, normal life." (FGD3, P5)

As participants adapted to living with AF, which required daily medication and frequent INR tests, their ongoing emotions were variable. Many participants mentioned the sadness they felt due to the obligation of taking warfarin every day. Other participants spoke about being fearful and worried that their self-management of diet and food and daily activities were inadequate to avoid complications. One participant also expressed the annoyance of having to take regular INR tests, whilst another participant expressed their gratitude for warfarin despite any difficulties, limitations, or challenges that they experienced.

"I've never been on medication. I never liked it, not even for a headache. So, having to take this medicine every day, I am sad." (FGD1, P7)
"[taking warfarin] you're afraid of getting hurt and a lot of blood coming out, will I have a haemorrhage?" (FGD3, P4)

Theme 4: Medication regimen

The following four sub-themes resulted from the medication regimen theme: polypharmacy, medication change, side effects, and medication management (Table 2). Participants from all three FGDs spoke of comorbidities and how they take multiple medications in addition to warfarin. Due to this polypharmacy, some participants expressed the difficulties and uncertainties about when they should take all their medications. Whilst some participants expressed their acceptance of, and willingness to, live with their medication regimen, other participants did not understand why they must continue taking so many medications if their blood tests and examinations were good.

"I've been doing these treatments, taking various medications, Propafenone, which is for the heartbeat, I take the medication for diabetes, Metformin, I take warfarin, I take blood pressure medicine, the blood fat medicine… but I started to get intrigued because my tests are excellent, everything I do, the only thing that show on the tests is the arrhythmia. I asked him why almost all the tests are good and I still have to take a lot of medicine, so he said they are good because of the medicine." (FGD3, P2)

Participants from all FGDs discussed their journey of medication change to get to where they are now. Most participants were on warfarin at the time of the FGDs; however, a few participants had previous experience with NOACs for the management of AF. Participants preferred taking NOACs in terms of not having to take INR tests, but often they could not afford NOACs as they are not offered free of charge in Brazil and therefore, they were forced to switch to taking warfarin. Several participants spoke about their previous experience with aspirin and how it was not effective or made them feel unwell which finally led to them switching to use warfarin.

"But there are better medicines … Pradaxa [NOAC] they gave me, I didn't need to do [INR test], you take it, it's very expensive … I'm working and everything, but it's already difficult to pay … it's very difficult, medicine, it shouldn't have that price." (FGD3, P1)

Once on warfarin, participants from the second and third FGD discussed the many side effects they experienced and the many side effects they are now attentive to, such as any signs of bleeding or clinical changes. Participants expressed their difficulties in adjusting and adhering to taking warfarin, but most accepted that it was part of their day-to-day life now. Many participants expressed difficulty in understanding how to take warfarin, others said they were unable to monitor the INR because the tests are unavailable in the nearest health facility and many-faced problems with dietary restrictions due to warfarin.

"Veins started to appear here in the left leg, bruises, this is visible, it really bothers me, if I put my hand on my leg it hurts, it becomes sensitive in the leg, in addition to being visible, this anticoagulant problem, which is a good and bad thing, if I don't take it anymore it can form a clot, give a heart attack, or a stroke, but I need the warfarin, I need the anticoagulant, if it doesn't thin the blood, it won't be able to help keep my heart balance its beat, so you see, those are unpleasant symptoms I'll have." (FGD3, P4)

Medication management can often be a challenge especially for elderly patients; however, participants' fear of forgetting or taking the wrong dosage led to them developing several strategies to remind themselves: some used an alarm clock to remind them of the medication; some separated the dosages in a box that identifies the time of day that each tablet should be taken; and some set reminders in strategic places. Despite these tactics, a few participants admitted to forgetting their dose or not taking the correct dose but expressed that it is something they try hard to avoid.

"Veins started to appear here in the left leg, bruises, this is visible, it really bothers me, if I put my hand on my leg it hurts, it becomes sensitive in the leg, in addition to being visible, this anticoagulant problem, which is a good and bad thing, if I don't take it anymore it can form a clot, give a heart attack, or a stroke, but I need the warfarin, I need the anticoagulant, if it doesn't thin the blood, it won't be able to help keep my heart balance its beat, so you see, those are unpleasant symptoms I'll have." (FGD3, P4)

"If you forget once in a while … that's fine. So, for example, it has already happened to me: I went to take a shower, took my shower, laid in bed and the reminder came, then I was in doubt, did I take the medicine? As I have a stepdaughter who is a doctor, I called her saying that I had forgotten to take all my medications, she said that everything was fine and that tomorrow morning you will take them but avoid not forgetting." (FGD3, P2)

Comment #7: *There appears to be some misreferencing on page 20 of 32. Reference 24 to 26 mentioned in line 18 are methodology references rather than included studies and Line 20 references 35 and 38 appear to be the same, and a reference relating to China in the 35,38,39 group missing. It would be good to check all reference citations are accurate.*
The claim, in Line 22 on the same page, about Healthcare professionals is supported by a reference about healthcare professionals in Brazil, so this restriction should be mentioned, or references from other countries added to support the claim.

Reply to Comment #7: We have corrected these references/text in the discussion (see below) and confirm that we have checked all other references throughout the manuscript.

"Similar findings have been reported from other qualitative studies conducted in high-income countries with people with AF and their caregivers.[34,35,35,37] From previous pathway mapping of AF care in Brazil,[39,42] Sri Lanka[44] and China (under review), access to and regular need for INR tests were reported as barriers to optimal AF care. Furthermore, a previous study reported that primary care clinicians often feel less confident about prescribing and monitoring warfarin due to uncertainties about bleeding risk. Healthcare professionals in Brazil have indicated their preference for prescribing NOACs over warfarin for the majority of people with AF[44] and NOACs could reduce the need for INR tests and therefore reduce visits to healthcare facilities which for many people in LMICs require long distance to travel." (Page 22, lines 16-25)

Reference 34: Bajorek BV, Ogle SJ, Duguid MJ, et al. Balancing risk versus benefit: the elderly patient's perspective on warfarin therapy. Pharm Pract (Granada). 2009;7(2):113-23.
Reference 35: Bajorek BV, Ogle SJ, Duguid MJ, et al. Management of warfarin in atrial fibrillation: views of health professionals, older patients and their carers. Med J Aust. 2007 ;186(4):175-80.
Reference 36: Bartoli-Abdou JK, Patel JP, Crawshaw J, et al. Exploration of adherence and patient experiences with DOACs one year after switching from vitamin-K antagonists- insights from the switching study. Thromb Res. 2018 ;162:62-68.
Reference 37: Dantas GC, Thompson BV, Manson JA, et al. Patients' perspectives on taking warfarin: qualitative study in family practice. BMC Fam Pract. 2004 ;5:15.
Reference 39: Goulart AC, Varella AC, Gooden TE, Lip GYH, Jolly K, Thomas GN, Lotufo PA, Greenfield S, Olmos RD, Bensenor IM, Manaseki-Holland S; NIHR Global Health Research Group on Atrial Fibrillation Management. Identifying and understanding the care pathway of patients with atrial fibrillation in Brazil and the impact of the COVID-19 pandemic: A mixed-methods study. PLoS One. 2023;18(10):e0292463.
Reference 42: Goulart AC, Varella AC, Gooden TE, Lip GYH, Jolly K, Thomas GN, Lotufo PA, Greenfield S, Olmos RD, Bensenor IM, Manaseki-Holland S; NIHR Global Health Research Group on Atrial Fibrillation Management. Identifying and understanding the care pathway of patients with atrial fibrillation in Brazil and the impact of the COVID-19 pandemic: A mixed-methods study. PLoS One. 2023;18(10):e0292463.
Reference 43: Sheron VA, Shanmugathas S, Gooden TE, Guruparan M, Kumarendran B, Lip GYH, Manaseki-Holland S, Nirantharakumar K, Shribavan K, Subaschandren K, Haniffa R, Surenthirakumaran R, Thomas GN, Uruthirakumar P, Greenfield S, Lane DA, Beane A; NIHR Global Health Research Group on Atrial Fibrillation Management. Healthcare provider and patient perspectives on access to and management of atrial fibrillation in the Northern Province, Sri Lanka: a rapid evaluation of barriers and facilitators to care. BMC Health Serv Res. 2022;22(1):1078.
Reference 44: Paschoal E, Gooden TE, Olmos RD, Lotufo PA, Benseñor IM, Manaseki-Holland S, Lip GY, Thomas GN, Jolly K, Lancashire E, Lane DA. Health care professionals' perceptions about atrial fibrillation care in the Brazilian public primary care system: a mixed-methods study. BMC Cardiovascular Disorders. 2022 Dec ;22(1):559.

Comment #8: *Some of the strengths and limitations mentioned, such as small sample sizes, are integral to qualitative research. One of the limitations not mentioned is that the people were from one area of Brazil so the results may not reflect other areas of Brazil or other countries.*

Reply to Comment #8: Thank you for this suggestion. We have added this additional limitation to our discussion (see below).

"*Although participants were from one area of Brazil, thus limiting transferability, many of our findings align with research from existing evidence (including in high-income countries), indicating that the care and support desired may represent a universal need."* (Page 22, lines 4-7)

Comment #9: *The conclusion should directly address the aim/objectives of the study, with the other ideas, particularly the first sentence of the current conclusion, incorporated into the discussion.*

Reply to Comment #9: Thank you for this suggestion. We have now added the study's aim within the first sentence of the conclusion.

"This qualitative study aimed to provide insight on how patients with AF cope with living with the condition in Brazil to inform services and healthcare delivery from an LMIC setting about the

appropriate support patients require to optimise patients' quality of life and wellbeing. Our findings highlight that people with AF often feel shocked and worried about their initial diagnosis due to lack of knowledge about AF, feel burdened with the inconvenience of monthly INR tests when on warfarin, feel worried about how to take warfarin in conjunction with other medications, and forced to take warfarin due to the inaccessibility of NOACs and uncertainty regarding daily lifestyle behaviors, such as diet. Improvements in public knowledge about AF, accessibility of NOACs, and improved care and support for people with multiple chronic conditions may be warranted to aid people with AF cope better with living with this chronic condition in a way that reduces stress and optimises wellbeing and quality of life." (Page 24, lines 9-20)

Comment #10*: How people cope with AF is a valuable topic; thank you for investigating it.*

Reply to Comment #10: We appreciate your valuable suggestions and thank you for the kind comments about the importance of our work.
